# Supplementary figures and images for: Current freezing and thawing scenarios employed by North Atlantic fisheries: their potential role in Newfoundland and Labrador’s northern cod (Gadus morhua) fishery
Source: PeerJ. 2021 Dec 10;9:e12526. doi: 10.7717/peerj.12526 (PMC8667752; doi:10.7717/peerj.12526)

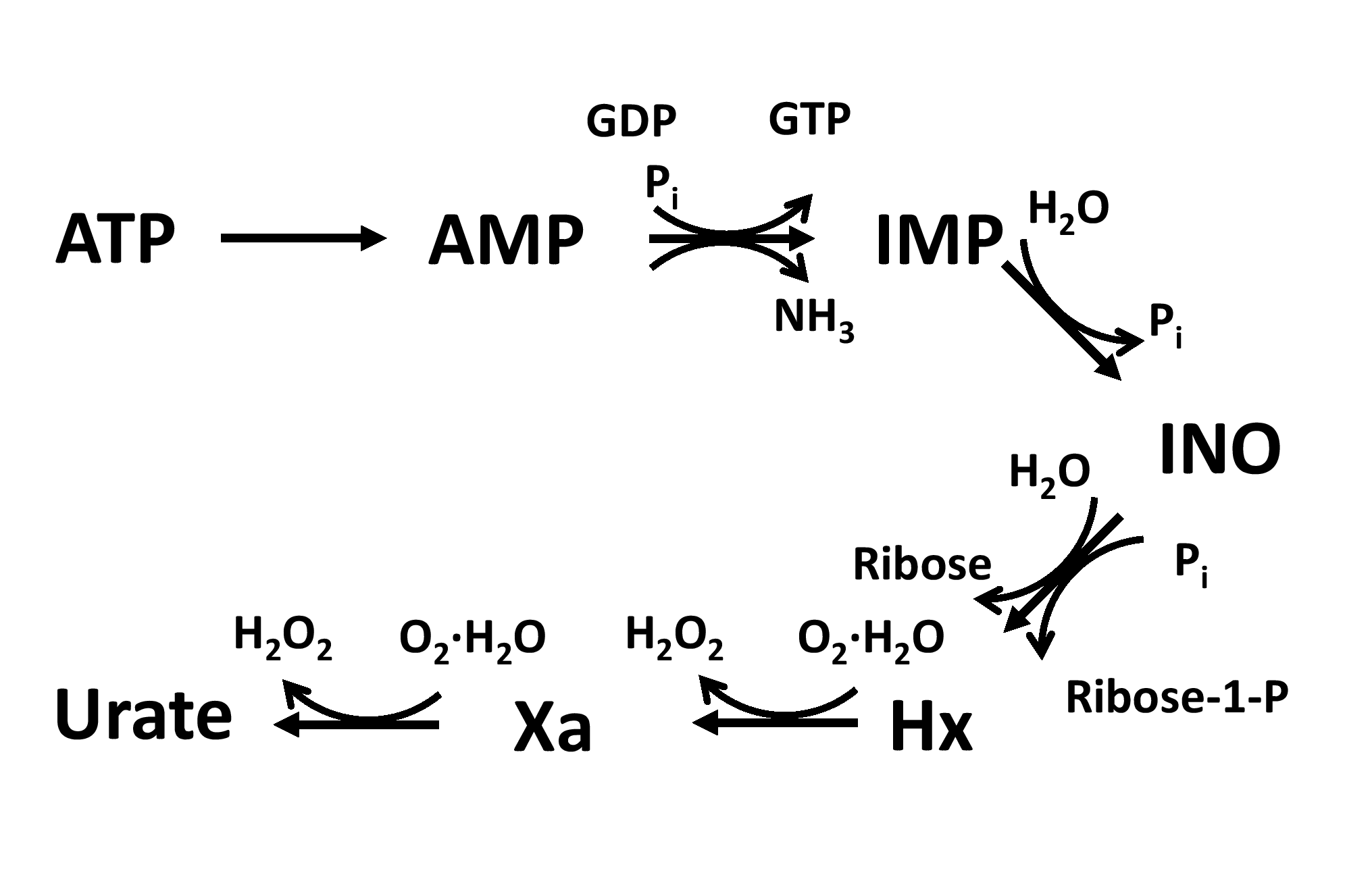

Supplement: Supplemental Information 1 — Source : (Huss et al., 1995; Surette, Gill & LeBlanc, 1988) [file peerj-09-12526-s001.png]

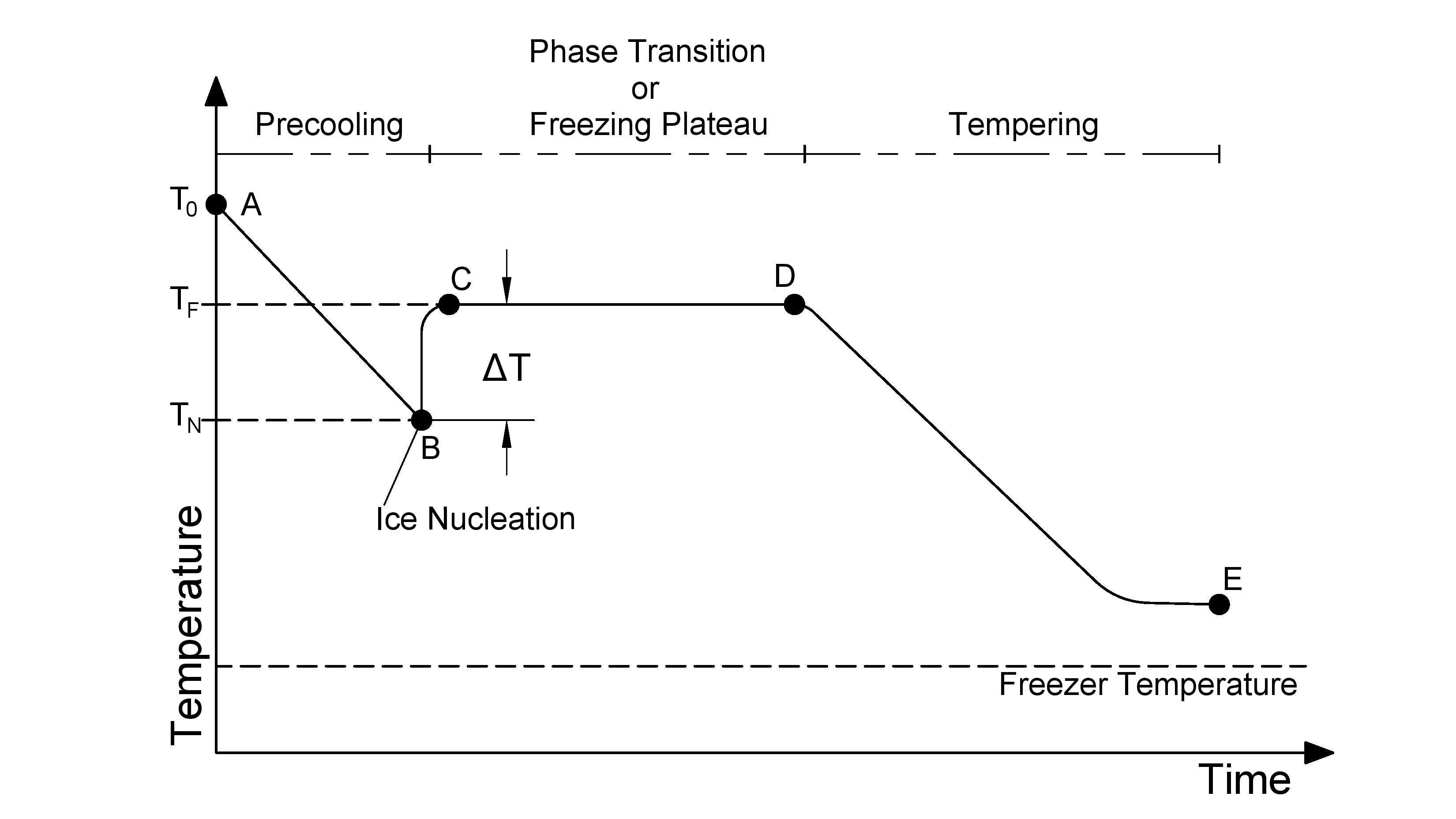

Supplement: Supplemental Information 2 — T0 - initial product temperature, TF –freezing temperature, TN –nucleation temperature, ΔT –supercooling temperature Source: (Otero et al., 2016) [file peerj-09-12526-s002.png]

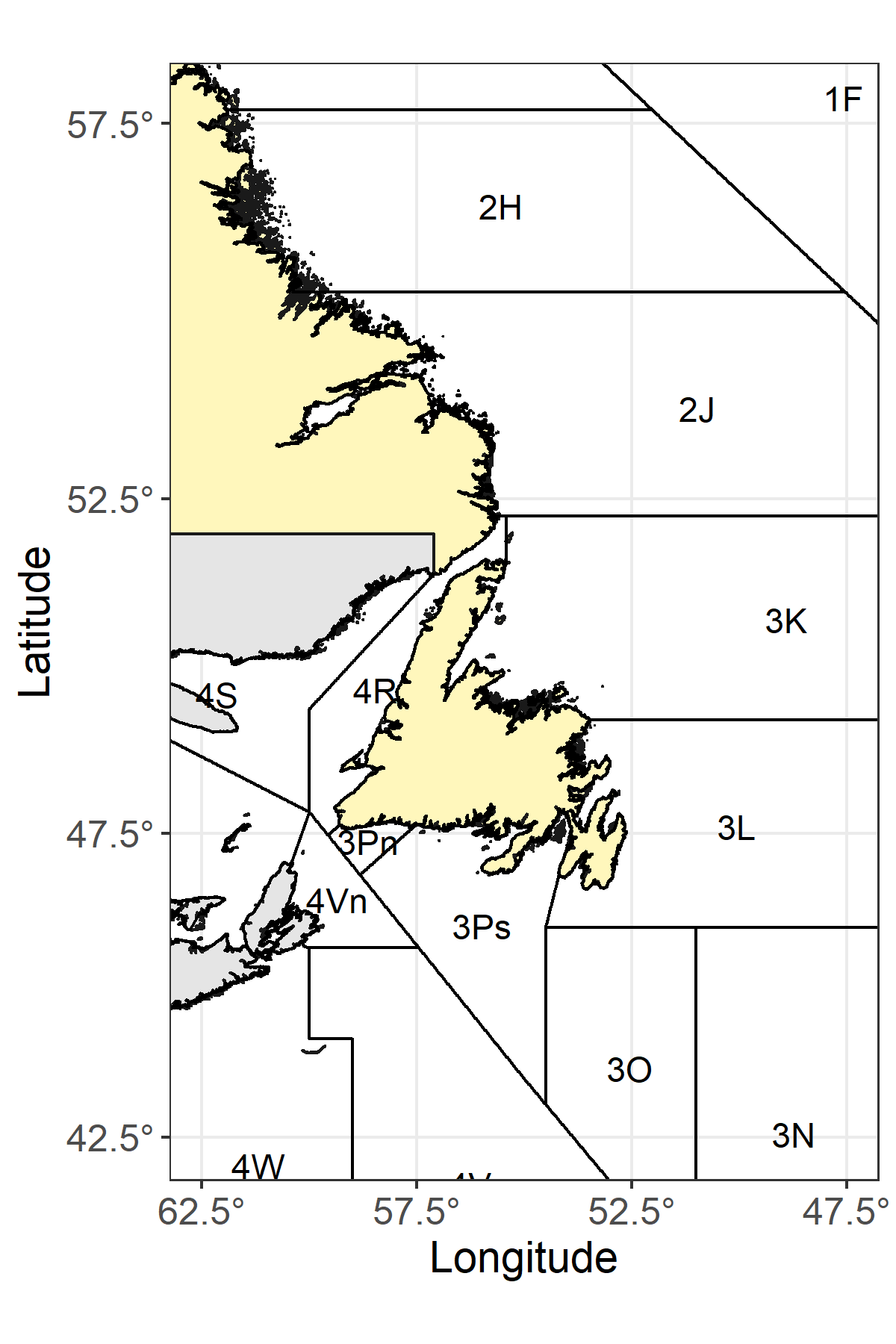

Supplement: Supplemental Information 4 [file peerj-09-12526-s004.zip › Fig_23.png]
